# Predicting 7‐year‐olds mental health in the perinatal period: Development and internal validation of a multivariable model using the prospective ALSPAC cohort

**DOI:** 10.1002/jcv2.70091

**Published:** 2026-01-03

**Authors:** Emma Butler, Michelle Spirtos, Linda M. O’Keeffe, Mary Clarke

**Affiliations:** ^1^ Department of Population Health Royal College of Surgeons Ireland Dublin Ireland; ^2^ Department of Occupational Therapy Trinity College Dublin Dublin Ireland; ^3^ School of Public Health University College Cork Cork Ireland; ^4^ MRC Integrative Epidemiology Unit at the University of Bristol University of Bristol Bristol UK; ^5^ Population Health Sciences Bristol Medical School University of Bristol Bristol UK; ^6^ Department of Psychology and Psychiatry School of Population Health & Department of Psychiatry Royal College of Surgeons Ireland Dublin Ireland

**Keywords:** child mental health, perinatal period, prediction modelling

## Abstract

**Background:**

Mental health difficulties in childhood are increasing. Prevention is the only sustainable and ethical public health approach. However, predicting which children are most at‐risk of mental health difficulties prior to symptoms emerging remains elusive.

**Methods:**

We developed and internally validated a perinatal multivariable model, predicting 7‐year‐olds mental health, using the Avon Longitudinal Study of Parents and Children (*N* = 6021, 51.2% male, 98.6% White). Perinatal predictors were reported by the mother prospectively in pregnancy and the Strengths and Difficulties Questionnaire (SDQ) was completed by the mother at 7‐years‐old. This was dichotomised at recommended clinical cut‐off (total>16) Building on our previous model in a French cohort, 15 perinatal parameters spanning maternal pre‐pregnancy health, biological and psychosocial pregnancy‐specific‐experiences, maternal health behaviours in pregnancy and sociodemographic factors were entered into a logistic regression using the least absolute selection and shrinkage operator. Optimism‐adjusted estimates were achieved using bootstrapping. Model performance was stratified by sex, sociodemographic risk and admission to a special‐care baby unit.

**Results:**

Combining eight variables predicted poor mental health, with a C‐statistic of 0.66; 95% Confidence‐Interval (0.64–0.68). It accurately predicted 85.6% of the participants mental health at 7‐years in the perinatal period. Model performance was similar across groups of interest. Applying this model leads to a higher benefit than serving ‘all’ or ‘no’ children, that is, using the model, 30.9% of children who later had poor mental health would have been identified in the perinatal period.

**Conclusion:**

It is possible to predict childhood mental health at birth with moderate accuracy. Similar patterns of model performance were observed in this English cohort compared to a previous French cohort. At population‐level, the model is most useful for ruling‐out babies who are not predicted to be high‐risk. In addition to improving its positive predictive value and external validation, future research should examine the model's performance at service‐delivery level before implementation.

## Key Points

**What is known?**

- Prevention approaches are required to tackle the increasing rates of child mental health difficulties. Policies focus on early identification of poor mental health.

**What is new?**

- It is possible to predict 7‐year‐olds mental health with moderate accuracy at birth using routinely available information.

**What is relevant?**

- In practice, this model would work best in a tiered intervention approach by screening out low‐risk children, and then assessing high‐risk children further to determine the level of intervention required.
- In research, future authors and studies should aim to externally validate these findings. Agreeing risk‐threshold levels with relevant stakeholders would also be beneficial to further the field of prediction modelling in child mental health.

## INTRODUCTION

Worldwide, the estimated prevalences of mental health difficulties in childhood range from 10% to 20% (Kieling et al., 2011). However, these problems are often undetected, and frequently not treated (Weitzman et al., 2015) despite being responsible for significant morbidity and mortality in childhood (McGorry et al., 2024). Around 8% of five‐to‐nine‐year‐olds live with a diagnosed mental disorder (WHO, 2022). Aside from Autism Spectrum Disorder (ASD) and intellectual disability, anxiety disorders and Attention Deficit Hyperactivity Disorder (ADHD) are often the earliest disorders to emerge in the pre‐school and early school‐age years (Weitzman et al., 2015). However, even greater numbers of children have problems causing impairment that do not meet criteria for a DSM‐5 disorder (Weitzman et al., 2015). After their onset, mental disorders often persist, disrupting the capacity for young people to fulfil their potential (Fusar‐Poli et al., 2021).

The need for care precedes the emergence of a traditional diagnostic picture (McGorry et al., 2024). Policies focus on early identification of poor mental health (Government of Ireland, 2019) but do not specify how to achieve this. Risk‐stratifying families at an early stage to receive preventative interventions or extra support may provide protection against later poor mental health.

A clinical prediction model (Steyerberg, 2019) aims to predict outcomes in individuals to support medical decision‐making (la Roi‐Teeuw et al., 2024). Predicting mental health symptoms in childhood lends itself to the development of a risk calculator because adult mental health has its roots in early life, the adverse consequences of poor mental health are known, early intervention has the potential to improve/delay illness onset and there is substantive evidence to support possible candidate predictors (Caye et al., 2019). For example, there is good evidence that pre‐ and peri‐natal maternal stressors are associated with increased risk of child mental health outcomes (Askelund et al., 2023; Butler et al., 2024; Debray et al., 2015; Frazier et al., 2023; Helmikstol et al., 2023; Kirkbride et al., 2024; Tien et al., 2020). A strong linear relationship between increasing number of psychosocial risks and mental health in childhood has also been demonstrated (Butler et al., 2024; Larson et al., 2008; Ostergaard et al., 2016; Wallander et al., 2019).

Novel biomarkers such as brain structure and function have been investigated, but as yet have not proven useful in predicting mental disorders (MacNeill et al., 2021). But this affords the opportunity to discover more useful prognostic factors. The first 1001 days is a prime opportunity for prevention as it is already a period of more intensive contact with healthcare professionals (Black et al., 2017; National Research Council, 2000; Nelson, 2000; Nelson et al., 2023) and the most frequent area of developmental delay identified in infancy is in gross‐motor development (Valla et al., 2015). It is known that motor coordination difficulties frequently co‐occur with mental health problems in clinical populations (Hill et al., 2016; Lee et al., 2020; Rigoli & Piek, 2016). A systematic review suggests that motor impairment is highly prevalent in children and adolescents with ASD and ADHD but there is a paucity of research relating to children with disruptive behaviour disorder or depression (Van Damme et al., 2015). And few studies have examined the prognostic potential of motor development (Katagiri et al., 2021; Pant et al., 2022; Piek et al., 2010). Whether this association between motor coordination and mental health holds in the general population and/or whether motor difficulties are prognostic of later mental health beyond neurodevelopmental disorders (Pant et al., 2022) is unknown.

Most models in mental health have been developed on adult samples and have not been externally validated in independent samples (Meehan et al., 2022), with a model predicting depression at 18‐years‐old from information known at 15‐years‐old (Rocha et al., 2021) being a notable exception. Whilst prediction models for different childhood mental health outcomes such as self‐harm (King, Grupp‐Phelan, & Brent, 2019), future diagnosis (Koning et al., 2019) and functional outcomes (Latham et al., 2019) have been developed, a systematic review (Senior et al., 2021) concluded that none of the existing models in the field of child and adolescent mental health (CAMHS) can be recommended as most were not sufficiently powered or calibration tested. Furthermore, none of the reported 100 models in the CAMHS review were designed to be applied in the perinatal period in the general population. In response to this gap, we previously developed and internally validated a perinatal model predicting child mental health at 5‐years using a French cohort (Butler et al., 2025a, 2025b). With a C‐statistic of 0.67; 95%Confidence Interval (CI) (0.64–0.69), 10 perinatal variables predicted mental health at 5‐years. Using a risk‐threshold of ≥8% to define low and high‐risk cases, it correctly classified 78.8% of children.

External validation studies are an important but neglected part of prediction model research (Collins et al., 2015; Riley et al., 2024) and such studies should apply the model as originally specified and quantify the accuracy of the predictions made, with a focus on reproducibility or transportability (Riley et al., 2024). At a minimum, an external dataset must contain the outcome of interest and the values of any predictor in the original model (Riley et al., 2024). Should these be unavailable, model work is then considered to be the development of a new model. We had intended to externally validate the French model to assess its transportability but as two predictors were unavailable in the ALSPAC dataset, we were unable to achieve this. Rather than developing an entirely new model with no commonalities to the original model, we imitated the original model as far as possible by using a similar population, similar predictors that were available, the same start‐point, the same outcome (albeit a slightly later timepoint) but in a different temporal and geographical setting. Although not external validation, it will aid our understanding of the potential performance of a similar model in a different location and time (Van Calster et al., 2023).

Therefore, this study aims to: (1) develop a prediction model for risk of poor mental health at 7‐years‐old using data in the perinatal period (2) examine the model's stability at population and individual‐level (3) examine the performance of the model at pre‐specified risk‐thresholds of 8%, 15% and 25% and its fairness and accuracy in subgroups of interest (sex, sociodemographic risk and whether baby was in a special care baby unit [SCBU]). A secondary aim was to ascertain if fine‐ or gross‐motor difficulties in infancy were prognostic of later mental health.

## MATERIALS AND METHODS

### Study design

Data were from first‐generation children of the Avon Longitudinal Study of Parents and Children (ALSPAC). This is a population‐based prospective pregnancy cohort study from southwest England, with details of the design, sample and measures used presented elsewhere (Boyd, Golding, .., & Henderson, 2013; Fraser et al., 2013). In brief, pregnant women resident in Avon, UK with expected dates of delivery between 1^st^ April, 1991 and 31^st^ December, 1992 were invited to participate. ALSPAC enroled 14, 451 pregnancies, from which 14,062 live‐births occurred with 13,988 children alive at 1‐year‐old. Ethical approval for the study was obtained from the ALSPAC Ethics and Law Committee and the Local Research Ethics Committees. Informed consent for the use of data collected via questionnaires and clinics was obtained from participants following the recommendations of the ALSPAC Ethics and Law Committee at the time. The ALSPAC website contains details of all the data that is available through a fully searchable data dictionary and variable search tool (https://www.bristol.ac.uk/alspac/researchers/our‐data). This study uses data gathered in pregnancy, at birth, and at 7‐years‐old. This secondary data analysis was approved by the Research Ethics committee for the Royal College of Surgeons Ireland (RIMS 212610659).

### Measures

#### Candidate predictors from the ELFE model

The perinatal predictors in the French ELFE multivariable model predicting child mental health at 5‐years (Butler et al., 2025a, 2025b) were considered as candidate predictors for this study. They were registered a priori on osf.io (https://osf.io/ae7hd/) and with consideration to the sample size calculation (Supporting Information S1: Table S1). These included (1) maternal pre‐pregnancy health: gravidity, mental health, history of blood pressure problems (2) biological and psychosocial pregnancy‐specific experiences (PSEs), (3) smoking and drinking alcohol in pregnancy, (4) whether baby had been in a special care baby unit, infant sex and (5) post‐natal cumulative sociodemographic risk (SR) (Table 1 and Supporting Information S1: Table S2).

#### Maternal pre‐pregnancy health

These were all self‐reported by the mother at ∼12/18‐weeks gestation.

*Gravidity*—have you been previously pregnant?

*Maternal mental health*—have you ever experienced drug addiction, alcoholism, schizophrenia, anorexia, severe depression or ‘other’ psychiatric problems?

*History of blood pressure problems*—have you previously had high blood pressure (outside of, or during, a previous pregnancy)?

#### Pregnancy Specific Experiences (PSEs)

##### Biological PSEs

The following items were self‐reported by the mother at ∼18‐weeks‐gestation (relating to trimester 1), 32‐weeks‐gestation (for trimester 2) and ∼8‐weeks post‐natal (for trimester 3): nausea, vomiting, diarrhoea, bleeding, urinary tract infection, flu, rubella, thrush, herpes, ‘other’ infection, injury/shock, diabetes (existing, gestational or glycosuria), high blood pressure (existing or new occurrence after 20 weeks gestation) and ‘other’ complication. Number of conditions experienced across pregnancy were summed for each woman. The maximum possible count was 14 (i.e., one point for each item experienced at ANY time during the pregnancy) with the maximum experienced by any participant being 10 (S2). This was categorised into four groups as follows: 0 (endorsed 0 or 1 experiences), 1 (endorsed 2, 3 or 4 experiences), 2 (endorsed 5 or 6 experiences), and 3+ (endorsed 7–10 experiences) (S.2 & T.1).

##### Psychosocial PSEs

This was an existing constructed variable developed and supplied by the ALSPAC study team referred to as family adversity index. It consists of 18 items measured at ∼8, 12, 18 and 32‐weeks‐gestation using standardised and non‐standardised questionnaires. It includes information about housing, finances, relationship quality, mental health, social network, substance abuse and involvement with crime. They categorised this as none (no adversity), mild (one or two adversities) and severe (>2 adversities) experienced in pregnancy. For specific details (Winsper et al., 2012) and S.2.

*Smoking in pregnancy*—mother self‐reported on a questionnaire (completed between eight and 42‐weeks gestation) the number of cigarettes that she smoked at that time. We dichotomised as none v any.

*Drinking alcohol in the first trimester of pregnancy*—mother self‐reported on a questionnaire (measured ∼18‐weeks gestation), the amount of alcohol she consumed in the first trimester of pregnancy. We dichotomised as none v any.

*Baby had been in a special care baby unit* (binary yes/no)—self‐reported by the mother on a questionnaire at ∼ 4‐weeks post‐natal.

*Infant sex* (male/female) ‐ self‐reported by the mother on a questionnaire at ∼ 4‐weeks post‐natal.

#### Post‐natal cumulative SR

Aggregating multiple risk factors can more accurately ascertain the severity of socioeconomic adversity and has more predictive power than focusing on the effects of any single indicator (Evans, 2004; Evans et al., 2013). We constructed a SR score, that has previously been validated in pregnant women (Li et al., 2020) and that we have demonstrated associates with child mental health (Butler et al., 2024; Butler et al., 2025a, 2025b) in Irish and French populations, based on post‐natal sociodemographic characteristics of the child's environment at 1‐month old (S.2) that were self‐reported by the mother on questionnaires administered between 8‐weeks gestation and 4‐weeks post‐natal. It comprised maternal age, race, Townsend‐area‐level income quintile, maternal education and relationship status (Li et al., 2020). Both higher (36+years) and lower maternal age at birth (<26 years) were considered risks. Race was dichotomised as ‘white’ and ‘other’ with ‘white’ considered less risk. A 3‐category variable was created from Townsend income quintiles to denote high (Q4&5), middle (Q3) and low income (Q1&2). Maternal education was categorised as (1) no formal education/CSE (2) O‐level or vocational, (3) A‐level and (4) degree. Lower educational attainment corresponded to higher risk scores. Lastly, having a partner was considered low risk, and not having a partner considered higher risk. Values were then combined and divided into four sociodemographic risk categories: none, low, moderate and high.

#### Motor development in infancy

The child's fine‐ and gross‐motor development Z‐scores based on the Denver developmental assessment, administered at 18‐months‐old, completed by the mother were added as possible candidate predictors. Z‐scores were calculated by the ALSPAC study team and were available as constructed age‐adjusted variables with a mean of 0 and standard deviation of 1. We also categorised this variable into standard deviation groupings.

#### Outcome measurement

The Strengths and Difficulties Questionnaire (SDQ) was completed by the mother when the child was 7‐years‐old, as a measure of child mental health and well‐being (A. Goodman & Goodman, 2009). The SDQ is a valid and reliable instrument to screen for emotional and behavioural problems in children aged 3–16 years and is widely used in research and clinical practice (Dachew et al., 2023). It is a parent‐rated questionnaire containing 25‐items on a 3‐point likert scale (0 = not true; 1 = somewhat true; 2 = certainly true), five items are reverse scored. Item scores are aggregated into 5 subscales. The first four subscales combine to calculate a total score ranging from 0 to 40. Higher scores indicate higher difficulties. The SDQ‐total was dichotomised at the recommended cut‐offs (sdqinfo.org). Total scores of 17 or above were considered to be in the clinical range indicative of poor mental health. Parent‐reported SDQ‐total scale has higher internal consistency (Cronbach's‐alpha = 0.82) and test‐retest reliability than the four subscales (R. Goodman, 2001).

### Data analysis

We examined unadjusted associations between each categorical predictor and outcome using logistic regression (Supporting Information S1: Table S3). Next we inputted 15 **candidate** perinatal predictors (each level of a predictor was counted) as per the sample size calculation (S.1), using Least Absolute Shrinkage and Selector Operator (LASSO logistic regression) for variable selection. Bootstrapping (1000 repetitions) was used to penalise for the known optimism of prediction models in development data from over‐fitting to estimate optimism‐corrected performance (internal validation). This is estimated by using different data samples from the same underlying population.

The predicted probabilities generated from the model (without the motor variables) were compared with the observed outcomes using several performance metrics: (1) C‐statistic (AUROC) which ranges from 0 to 1 and gives the probability that for any randomly selected pair of participants, one with and one without the outcome, the model assigns a higher probability to the participant with poor mental health. A ‘good’ C‐statistic is ≥ 0.7 (Snell, Hua, Debray, & et al., 2016) (2) Calibration measures how well the predicted outcome of the model agrees with the observed outcome on average. This was assessed for each 10th of predicted risk, ensuring 10 equally sized groups through calculating the ratio of predicted to observed risk and plotting observed proportions versus predicted probabilities. (3) A perfectly calibrated model has a slope = 1. Slope <1 indicates over‐ whilst slope >1 indicates under‐fitted respectively (Steyerberg & Vergouwe, 2014). A ‘good’ calibration slope lies between 0.9 and 1.1 (Snell et al., 2016) (4) For calibration‐in‐the‐large (CITL), CITL<0 indicates predictions are too high. (5) Brier score is an extension of the mean square error for binary outcomes, comparing observed outcomes with estimated probabilities. Brier score ranges from 0 to 25 with lower values indicating better fit (Brier, 1950).

We examined the following instability plots (Riley & Collins, 2023): prediction, calibration and classification (using ≥8% threshold). We also calculated the average mean absolute prediction error (MAPE).

A risk‐threshold, typically based on clinical importance, is used to classify individuals into low and high‐risk groups. A traditional method for operationalising clinical utility is by evaluating positive predictive value (PPV) and negative predictive value (NPV) (Trevethan, 2017). Sensitivity, specificity, PPV and NPV were calculated at pre‐registered risk‐threshold points of 25%, 15% and 8% as no standard criteria for defining a risk‐threshold exists for the prediction of childhood mental health and we used these cut‐offs previously (Butler et al., 2025a, 2025b). Sensitivity relates to how well the model can classify children who truly have poor mental health. PPV, on the other hand is the proportion of children predicted to be high‐risk that had poor mental health at 7‐years (Monaghan et al., 2021). Whilst PPV is the usual test of performance, it can inaccurately reflect test performance when the overall base prevalence is low (Monaghan et al., 2021). Therefore, clinical utility was also evaluated using decision‐curve analysis (Vickers & Elkin, 2006).

We examined the prevalence of poor mental health across our groups of interest and compared whether there was evidence of differential performance across groups (Grote & Keeling, 2022) of interest (sex, sociodemographic risk and whether child was in a special care baby unit (SCBU)) by examining the models performance using the ‘roccomp’ function in STATA (StataCorp, 2021).

For our secondary aim to examine whether motor development is a prognostic factor, we repeated the modelling process but added the two motor variables as candidate predictors.

As a sensitivity analysis we examined whether the babies predicted group (i.e., predicted to be low‐ or high‐risk), based on an 8% risk‐threshold cut‐off predicted their mental health, later in childhood, at nine and 11‐years‐old. We also ascertained whether the prediction model performed any better than predictions based on singular known risk factors, that is, firstly cumulative sociodemographic risk and secondly history of maternal mental health problems.

### Participants in the analysis

Participants that had complete SDQ outcome data at age 7‐years (*n* = 8351) and complete data on all candidate predictors (*n* = 6021) were included in this analysis (Supporting Information S1: Figure S1).

### Missing data

We examined characteristics of participants included in our model versus those excluded from the analysis due to missing predictor or outcome data. We also examined the distribution and patterns of missing predictor data.

Statistical analysis was performed with STATA v.17 (StataCorp, 2021). PROBAST questions pertaining to participants, predictors and outcomes were considered in determining the risk of bias in using ALSPAC dataset for model development (Supporting Information S1: Table S4). Case‐mix distributions in the original French ELFE model and the current ALSPAC model were also compared (Supporting Information S1: Table S5). TRIPOD guidelines (Collins et al., 2015) for reporting were followed.

## RESULTS

Participants excluded from the analysis (Supporting Information S1: Figure S1) due to loss to follow‐up (*n* = 5582) or missing some candidate predictors (*n* = 2330), had slightly higher levels of: children experiencing poor mental health, smoking in pregnancy, babies in SCBU, adverse psychosocial pregnancy‐specific‐experiences and cumulative sociodemographic risk (Supporting Information S1: Table S6a). Of the 8351 that participated at 7‐years, 11% were missing one predictor, 11% were missing two predictors, 3% were missing three with 1.5% or less participants missing 4+ variables (Supporting Information S1: Table S6b). The variable that was most frequently missing was the cumulative sociodemographic risk variable (Supporting Information S1: Table S6c). *N* = 6021 thus had complete information and were included in the analysis.

Of the *n* = 6021 participants, *n* = 304 (5.1%) had poor mental health at 7‐years. Baseline characteristics are summarised in Table 1. 69.6% of the sample experienced none to low sociodemographic risk, 81.4% experienced none or very few biological pregnancy‐specific‐experiences with almost half (46.7%) experiencing no adverse psychosocial pregnancy‐specific‐experiences. 51.2% of the babies were male and the majority of mothers (65.4%) had previously been pregnant. 5.3% of babies spent time in SCBU. 71% of the sample had fine‐motor Z‐scores between +/−1 standard deviation (SD) of the mean at 18‐months. More children (88.4%) had gross‐motor Z‐scores between +/−1SD of the mean. 2.9% and 2.6% had fine and gross‐motor Z‐scores respectively more than ‐2SD below the mean (T.1).

**TABLE 1:** Summary of descriptive characteristics/predictors by outcome group.

	All % (*n* = 6021)	Adequate mental health % (*n* = 5717)	Poor mental health % (*n* = 304)
Maternal pre‐pregnancy health			
Gravidity (% yes)	65.4	65.5	63.8
Maternal mental health (% yes)	10.4	9.9	21.4
History of blood pressure problems (% yes outside pregnancy &/or during a previous pregnancy)	13.8	13.8	14.5
^a^Biological pregnancy specific experiences (%)			
0	15.6	15.8	12.2
1	65.8	66.0	62.8
2	16.6	16.3	22.0
3+	2.0	1.9	3.0
^b^Psychosocial pregnancy specific experiences (%)			
None	46.7	47.6	29.9
Mild	39.9	40.0	39.5
Severe	13.3	12.4	30.6
Maternal health behaviours in current pregnancy			
Smoked (% yes)	14.8	14.2	24.3
Drank alcohol in 1st trimester (% yes)	56.1	56.0	58.9
Birth/Post‐natal			
Special care baby unit (% yes)	5.3	5.2	6.6
Infant sex:			
Male (%)	51.2	50.7	60.2
^c^Sociodemographic risk (%)			
None	20.4	20.9	11.5
Low	49.2	49.2	49.0
Moderate	27.8	27.4	34.5
High	2.6	2.5	4.9
Motor development @ 18‐months			
Fine‐motor Z‐score mean (SD^d^)	0.02 (0.96)	0.05 (0.94)	−0.40 (1.2)
Gross motor Z‐score mean (SD^d^)	−0.02 (0.95)	−0.01 (0.95)	−0.12 (1.1)
Fine‐motor Z‐score categories (%)			
>+1 SD	14.4	14.6	10.2
1 to +1 SD	71.0	71.5	62.2
2 to −1 SD	11.7	11.3	18.8
≤‐2SD	2.9	2.6	8.9
Gross‐motor Z‐score categories (%)			
>+1 SD	0	0	0
1 to +1 SD	88.4	88.6	84.2
2 to −1 SD	9.0	8.8	12.5
≤‐2SD	2.6	2.6	3.3

^a^ Biological pregnancy Specific Experiences comprised number of medical illnesses/experiences from a possible list of 14 that specifically occurred during pregnancy.

^b^ Psychosocial pregnancy specific experiences comprised level of psychosocial adversity experienced specifically during the pregnancy from list of 18 items.

^c^ Sociodemographic risk comprised of maternal age, education level, relationship status, race and Townsend‐area‐level income quintile.

^d^ Standard deviation.

The prediction model for risk of poor mental health in childhood is presented in Table 2. Eight predictors (with 13 predictor parameters) were selected in the LASSO model: biological pregnancy‐specific‐experiences, psychosocial pregnancy‐specific‐experiences, cumulative sociodemographic risk, maternal mental health problems, gravidity, smoking and alcohol use in current pregnancy and infant sex. Discrimination (AUCs) did not change significantly after internal validation (Supporting Information S1: Table S7) using bootstrapping that is, optimism‐adjusted (AUC 0.66 (95% CIs: 0.64–0.68)). Calibration was good as evidenced by the calibration slope (1.02) and gradient. Calibration is over the entire range on average, when we looked at it by 10th deciles we can see that whilst our low risks were relatively accurately estimated, high predicted risks were over‐estimated. The optimism adjusted slope (S.7) stayed in the ‘good’ range but changed the interpretation from being under‐fitted to over‐fitted that is, after optimism adjustment, variation in estimated risks are too extreme, resulting in overestimated high risks and underestimated low risks (Austin & Steyerberg, 2019). The bootstrap uniform shrinkage factor is −0.10 (S.7).

**TABLE 2:** model performance and intercept and coefficients for the LASSO prediction model for childhood mental health (SDQ‐total >16) in children aged 7‐years (*n* = 6021).

Predictors	^a^Model coefficient
Intercept	−3.80
Gravidity (pregnant before)	−0.18
Maternal mental health problems (yes)	0.49
^b^Level of biological pregnancy‐specific	
0	Ref
1	0.15
2	0.40
3+	0.30
^c^Level of psychosocial pregnancy‐specific	
None	Ref
Mild	0.34
Severe	1.10
Smoked during the pregnancy (yes)	0.18
Consumed alcohol in the 1^st^ trimester in the pregnancy (yes)	0.06
Infant sex (female)	−0.31
^d^Cumulative sociodemographic risk	
None	Ref
Low	0.46
Moderate	0.51
High	0.71
Performance and discrimination metrics	
Discrimination & calibration	
Optimism adjusted AUC	0.66 (0.64–0.68)
Optimism adjusted calibration slope	0.93 (0.71–1.2)
E/O	1.00
Optimism adjusted CITL	0.001 (−0.17‐0.24)
Brier score	0.05

^a^ Coefficient from LASSO that is, includes shrinkage.

^b^ Biological pregnancy Specific Experiences comprised number of medical illnesses/experiences from a possible list of 14 that specifically occurred during pregnancy.

^c^ Psychosocial pregnancy‐specific‐experiences comprised level of psychosocial adversity experienced specifically during the pregnancy from list of 18 items.

^d^ Sociodemographic risk comprised of maternal age, education level, relationship status, race and Townsend‐area‐level income quintile.

The linear predictor was higher in children with poor mental health than in those without (Supporting Information S1: Table S8 and Figure S2). No child had a predicted risk higher than 0.26, the minimum predicted risk was 0.02 with median of 0.04 (Supporting Information S1: Figure S3).

**FIGURE 1:**
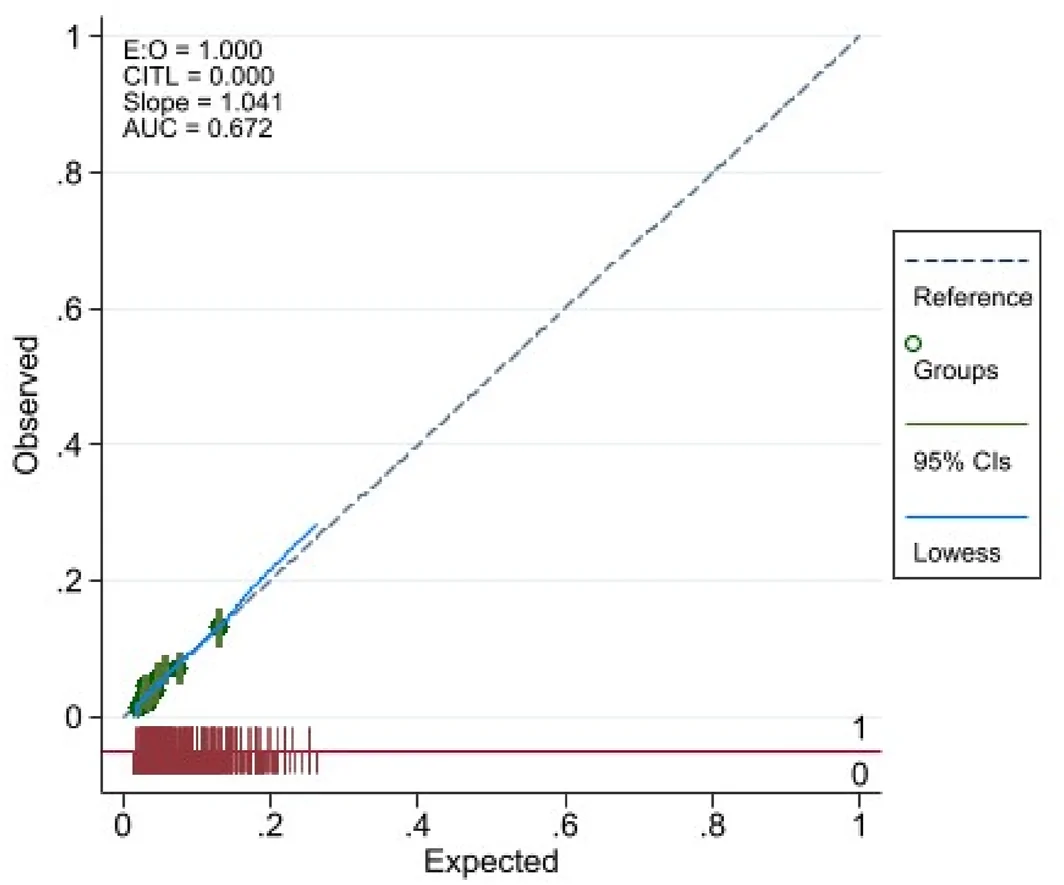
Calibration plot for the binary outcome model of poor mental health at 7‐years. Area below the dashed line = model's risk estimates are too high; area above the dashed line = model's risk estimates are too low. 10 circles = 10 groups defined by tenths of the distribution of estimated risks.

The calibration plot showed that predicted probability and observed frequency of poor mental health closely agreed in the 0%–20% range of risk. For calculated event probabilities beyond 0.2, the model under‐estimates the probability of poor mental health as evidenced by the smoothed calibration curve lying above the diagonal line (Figure 1).

### Stability

**FIGURE 2:**
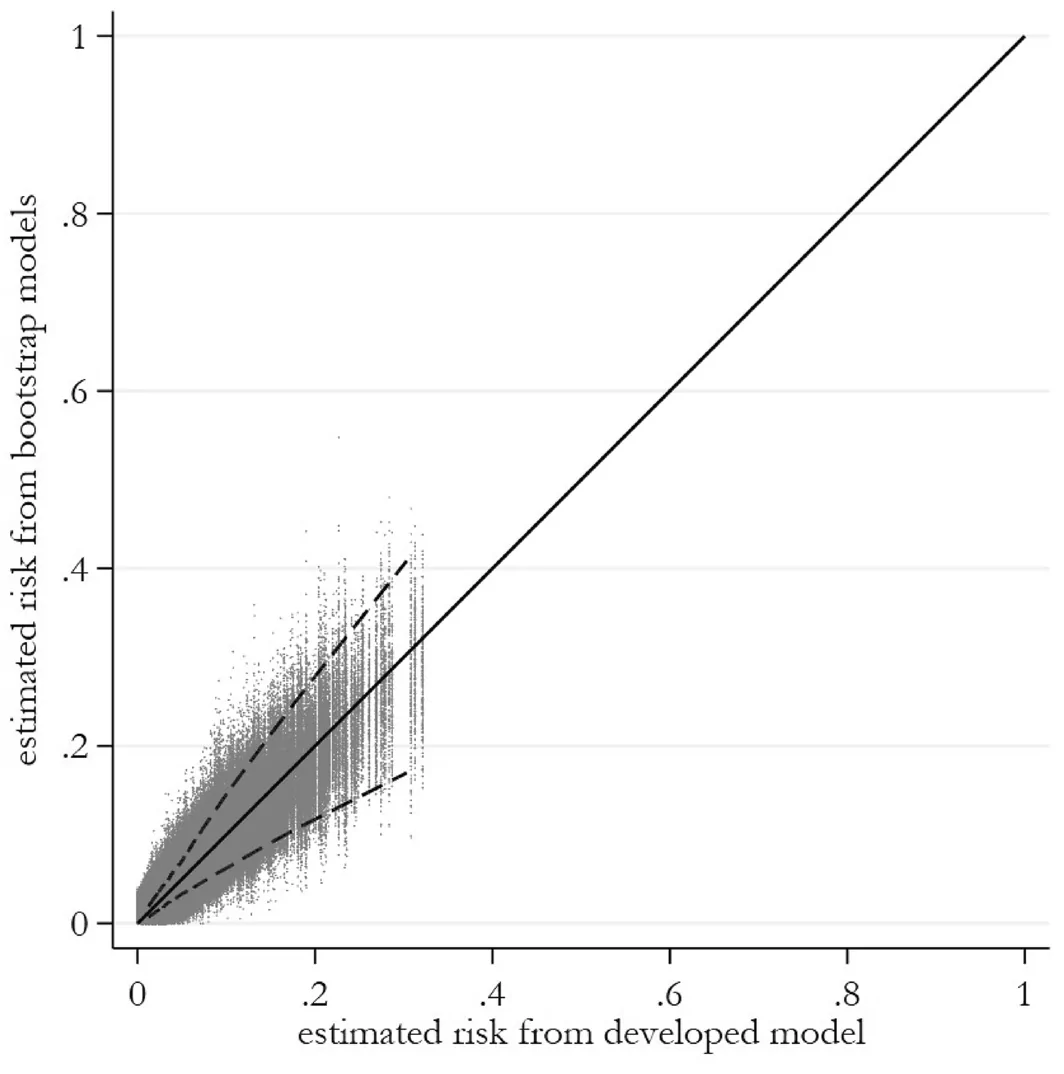
Prediction instability plot. Scatter of each individuals bootstrapped predicted values (*Y*‐axis) against their predicted value from the original model (*X*‐axis) and their 95% range.

Our model shows good stability at population‐ and individual‐levels across metrics. The prediction instability plot (Figure 2) is a scatter of each individuals bootstrapped predicted values (*Y*‐axis) against their predicted value from the original model (*X*‐axis) and their 95% range. Our plot shows that, for example, for a child with an original estimated predicted risk of 0.2, their bootstrapped 95% range of estimated risk is between 0.10 and 0.30. Defining our risk‐threshold for high‐risk at 0.08 (see thresholds below), their 95% range of predicted estimates are all within the high‐risk range.

For the calibration instability plot (Supporting Information S1: Figure S4a), the bootstrapped curves are overlaid on the same plot together with the calibration curve from the original model. The wider the spread of the bootstrapped curves, the greater concern for instability. Our calibration curves begin to deviate from the 45‐degree line once risk exceeds 0.18. (as our risk‐threshold is 0.08, all those categorised as high‐risk will still be high‐risk). This variability may still be reliable as they are still categorised the same overall. There is much less variability when the risk is < 0.18.

The classification plot (Supporting Information S1: Figure S4b) is a scatterplot of each individuals classification index (*Y*‐axis) plotted against their original predicted classification (*X*‐axis). Our plot has a narrow distribution with individuals values close to zero for most children except those with predictions close to the risk‐threshold (0.08).

In terms of discrimination, the C‐statistic instability histogram (Supporting Information S1: Figure S4c) shows small variability in C‐statistic estimates across bootstrapped samples (the majority all being between 0.65 and 0.675, with a tiny amount at 0.635 and 0.645). The average MAPE is 0.008. This means that, on average, across children, the absolute difference between their developed model prediction and the bootstrapped model's prediction is 0.008, thus instability in each individuals prediction is very low (Supporting Information S1: Figure S4d).

### Thresholds

Table 3 shows the percentage of children identified as at‐risk of poor mental health for different risk‐thresholds. Sensitivity, specificity, PPV and NPV were calculated at pre‐specified risk‐thresholds of 8%,15% and 25% based on our previous work.

**TABLE 3:** The categorisation of children as predicted ‘high’ or ‘low‐risk’ of poor mental health (SDQ‐total >16) at 7‐years based on the prediction model using different levels of risk‐thresholds compared to their observed outcome.

Proportion identified at high‐risk % (*n*)	Predicted ‘low‐risk’ group % (*n*)		Predicted ‘high‐risk’ group % (*n*)	
	Observed adequate mental health	Observed poor mental health	Observed adequate mental health	Observed poor mental health
@25% risk‐threshold				
	*N* = 6013		*N* = 8	
0.13 (8)	95.0 (5711)	5.0 (302)	75.0 (6)	25.0 (2)
@15% risk‐threshold				
	*N* = 5879		*N* = 142	
2.4 (142)	95.3 (5601)	4.7 (278)	81.7 (116)	18.3 (26)
@8% risk‐threshold				
	*N* = 5271		*N* = 750	
12.5 (750)	96.0 (5061)	4.0 (210)	87.5 (656)	12.5 (94)

Using a 15% risk‐threshold, the model identifies 2.4% of children at‐risk, with a sensitivity of 8.6%, specificity of 98.0%, PPV of 18.3% and NPV of 95.3% (T.3). As we reduce the risk‐threshold to 8%, the model identifies 12.5% of children at‐risk, sensitivity increases (30.9%), specificity decreases (88.5%), PPV decreases (12.5%) and NPV marginally increases (96.0%).

Whilst the 25% risk‐threshold had the best PPV (25.0%), in terms of absolute numbers, the 8% risk‐threshold (Supporting Information S1: Figure S5) had the highest sensitivity. That is, the highest number of children classified as being high‐risk who actually had poor mental health at 7‐years, *n* = 94/304 (30.9%). In terms of clinical utility, Supporting Information S1: Figure S6 shows this model has a net benefit for all thresholds <0.2, compared to treat‐all children or treat‐no children strategies.

### Subgroups

More males had poor mental health compared to females (6% vs. 4%) (Supporting Information S1: Table S9). The known dose‐response between sociodemographic risk groups and the proportion with poor mental health was evident (3%, 5%, 6% and 10% for none, low, moderate and high sociodemographic risk respectively). There was no significant difference in the proportion of children with poor mental health between those who did (6%) and did not (5%) spend time in the SCBU (S.9). There was no significant difference in the model's performance (AUC) between these groups of interest (Supporting Information S1: Table S10).

Finally, we included the child's fine‐motor and gross‐motor Z‐scores at 18‐months‐old as additional candidate predictors. The LASSO selected the fine‐motor variable as a predictor in the model (co‐efficient in Supporting Information S1: Table S11) showing that fine‐motor difficulties could be considered as a possible prognostic factor in future studies. This is also evident from Table 1 where the proportion of children with delayed fine‐motor development (Z‐score ≤ ‐2SD) was significantly higher in the children that later had poor mental health (8.9% compared to 2.6%). In fact, even somewhat delayed fine‐motor skills (Z‐score between ‐1SD and ‐2SD) was higher in the children that later had poor mental health (18.8% compared to 11.3%) The gross‐motor score was not selected by LASSO. Similarly, the proportion of children with delayed gross‐motor development was not significantly different between those who did and did not have later poor mental health (T.1).

As a sensitivity analysis, we examined the predictive ability of how the baby was categorised at birth (i.e., predicted to be high or low‐risk) on their mental health presentation at a later time‐point (nine and 11‐year‐old). Supporting Information S1: Table S12 shows that those predicted to be high‐risk at birth had an odds ratio of 3.6 (95%CI 2.6–4.9) for having clinical levels of poor mental health at 9‐years and an odds ratio of 3.0 (95%CI 2.1–4.2) for having clinical levels of poor mental health at 11‐years‐old compared to those predicted to be low‐risk at birth. Furthermore, we ascertained whether the prediction model performed any better than predictions based on singular known risk factors (cumulative sociodemographic risk and history of maternal mental health problems). Supporting Information S1: Table S13 shows that whilst all three models performed similarly in terms of the NPV, the full prediction model performed best in terms of PPV and sensitivity. 4.9%, 21.4% and 30.9% of children who experienced poor mental health were accurately identified at birth using sociodemographic risk only, history of maternal mental health difficulties only and the full prediction model discussed here respectively.

## DISCUSSION

Our analysis shows that it is possible to predict poor mental health at 7‐years using information known in the perinatal period with fair to moderate accuracy. We developed and internally validated a perinatal prediction model to inform potential preventative early intervention in the first 1001 days using similar predictors identified in a different cohort (ELFE) (Butler et al., 2025a, 2025b), other similarities were the start‐point, the same outcome but at a later time‐point and the target population that is, whole population. Evaluating the performance of an original model in new data (i.e., external validation) is an invaluable and crucial step prior to introducing a prediction model to routine clinical practice (Collins et al., 2015). Whilst external validation was not possible, (due to two variables not being measured in ALSPAC), we attempted to replicate findings. Of note, in the UK cohort from the 1990's used here, the model's performance was similar to that of the ELFE model, developed in a French cohort from 2011. The beta‐coefficients of the predictors included in both models were of similar magnitudes, therefore providing some support for the reproducibility of the model (Supporting Information S1: Figure S7). The overall discrimination performance of the models is in a similar range to each other and to other models in the field of child and adolescent mental health predicting future diagnosis later in the life course (Caye et al., 2019; Cohen et al., 2020; Koning et al., 2019; Moore et al., 2022; Rabelo‐da‐Ponte et al., 2020; Tate et al., 2020), however improving the model performance in terms of discrimination remains an important aim for future research. A well calibrated model produces predictions that on average closely align with the frequency of actual outcomes on aggregate. Strong calibration can be concluded if reliability holds across different patient subgroups. Poor calibration is known to be the Achilles heel for applicability of prediction models (Van Calster et al., 2019). The stability of this model is promising at individual and population‐level and it performs equally in subgroups of interest therefore despite its lower C‐statistic, this model should be built on that is, perhaps by adding another useful predictor rather than disregarded due to lower C‐statistic. Factors which are potentially contributing to the lower C‐statistic are: it is predicting a heterogenous outcome, there is a 7‐year timeframe (other models tend to have closer time‐points i.e. one‐2 years), there are no symptoms and no clinical markers included in the model which would substantially increase accuracy and are typically included in other models in the field (but we wished to predict difficulties before symptoms were evident). Furthermore, populations with a narrower case mix, that is, predictor values with more homogeneity in values across individuals, tend to have worse discrimination performance (Riley et al., 2016). Therefore, updating this model by adding a strong prognostic variable with a heterogeneous dispersion may improve the discrimination performance substantially (Janssen et al., 2008). The AUC of this model is still higher than other models developed for childhood mental disorder (0.60: 95%CI 0.58–0.62) (Green et al., 2022). In addition to the relatively low prevalence of the outcome, no‐one in this sample had a predicted risk >0.26 and so discrimination is poorer as patients have less extreme predictions (Binuya et al., 2022). Other models with better discrimination do not necessarily have adequate calibration and thus won't perform well in external validation and also would not be implementable in practice.

In addition to external validation of this ALSPAC model, future research should examine the models ‘local’ validation that is, at well‐baby checks before implementation, as a transportable model (i.e., a model that performs reasonably well in multiple populations/settings) may come at the cost of not providing the most accurate predictions feasible in a local setting. A good example of a well‐validated model is work conducted by the IDEA consortium which validated a prediction model to be used in adolescence to predict later depression in seven samples throughout the world (Piccin et al., 2025), however our focus is on prevention in the earliest years of life. Evidence‐based medicine guidelines recommend four steps in establishing a validated predictive tool and decision rules for use in clinical practice: (1) selecting variables (2) validation at a single site (3) validation at different sites (4) impact on clinical practice (Lawrie et al., 2019). Our (in)stability assessments provide reassurance that the prediction model is likely to be reliable at both population‐ and individual‐level, however, further validation of the model (in an external validation study) is still required.

Clinicians' ability to identify mental health problems in children in primary care, on the basis of clinical judgement alone, that is, without using a screening instrument has been shown to have low sensitivity, ranging from 14% to 54% but with specificities ranging from 69% to 100% (Sheldrick et al., 2011). This model performs best for stratifying children who *do not need* additional support, that is, the NPV rate. This is a known phenomenon, where the relatively low PPV reflects the low base rate of poor mental health in this population sample (in our case 5.1%). This means there is more certainty that a negative result indicates absence of outcome but there is less certainty that a positive test really indicates outcome presence (Altman & Bland, 1994; Lavigne et al., 2016). Of the children predicted to have a low‐risk of poor mental health, 96% of them do not have mental health difficulties at 7‐years. We are less confident in its ability to identify the children who are high‐risk as ‘definitely’ having poor mental health, as the PPV is lower that is, increased false positives. The purpose of this model is to identify babies who may benefit from closer monitoring/additional supports, as opposed to treating mental illness per se. As the focus is on prevention rather than correction of existing behaviours/difficulties, false positives may be less of a concern. A tiered intervention framework where high‐risk scores trigger a more holistic assessment rather than direct intervention, could help mitigate this limitation while ensuring appropriate resource allocation.

Furthermore, the PPV changes as prevalence increases, our prevalence is low at 5.1% so this model may perform more accurately in clinical samples where the prevalence is likely to be higher. For example, compared to other models whose accuracies were higher, the prevalences of the outcome were significantly higher (8.1%, 12% and 28.6%) (Caye et al., 2019) and (3.1%, 17.7% and 16.8%) (Rocha et al., 2021), to as high as 33% in a known high‐risk group, that is, those born very preterm/very low birthweight (Franz et al., 2022). Our sensitivity analysis shows, that babies predicted by the model to have a higher‐risk of poor mental health at 7‐years‐old had 3.6 and 3.0 times the odds of poor mental health at nine and 11‐years‐old respectively. For outcomes affecting <10% of the population, as is the case here, the odds ratio approximates the relative risk (Lovasi et al., 2012).

Additionally, the risk threshold cut‐point also impacts the PPV. That is, PPV improves as the risk threshold increases, but at the cost of the model under‐predicting more, that is, missing more children who actually ended up having poor mental health. In all subgroups, categorising risk at a 15% cut‐off resulted in predictions that were too low that is, underestimating high‐risk and missing many children, whilst categorising at 8% cut‐off resulted in predictions that were too high that is, over predicting the children at risk. Based on our findings we propose that future research should consider a risk‐threshold of 12% (Supporting Information S1: Table S14) as this appears to balance under and over‐predicting in each of our groups of interest that is, predictions most in line with observed values.

The intent of this model is to identify, at population‐level, the subgroup of children who may require provision of extra support, more holistic assessment or onward referral for early family interventions (such as family‐focused therapy, psychotherapy, parent education, home visiting etc). It would therefore be of interest to examine with families their level of perceived risk for availing of further supports (which likely could have some benefit to all children and families). It is likely that both families and professionals would accept lower risk‐thresholds as there is more concern about missing the opportunity to delay/prevent poor mental health in childhood than receiving unnecessary additional supports which would not cause harm.

Risk prediction models are used to inform individualised decisions about clinical actions therefore it is important they are robust and reliable for all users (Riley & Collins, 2023). We assessed model performance across sex, sociodemographic risk and SCBU given that under‐/over‐estimating risk for some groups could potentially lead to reduced access to preventative care or unfair burden respectively. Reassuringly, our model performance was similar across the three groups of interest.

Despite the prevalence and long‐term implications of early childhood behavioural difficulties they are rarely detected (Horwitz et al., 2003). If applied, this model would identify up to 4 true positives for every 100 children compared to no children in a do‐nothing strategy. Whilst this number seems small, at population‐level it would equate to a large absolute number of children identified before mental health difficulties are evident. There were 54,678 births in Ireland in 2023 (CSO.i.e.), thus 2187 infants would be identified by this model for further support who otherwise may not be identified as at‐risk. In classical decision theory, the strategy with the highest expected utility should be chosen irrespective of size or statistical significance. Theoretically, any improvement in net benefit is therefore worth having (Vickers et al., 2019).

We found that fine‐motor but not gross‐motor difficulties in infancy were prognostic of poor mental health at 7‐years in the general population. Future research should consider examining this relationship longitudinally, as, if early fine‐motor difficulties are prognostic of later mental health this enables us to identify and prioritise children who are referred for poor motor skills for mental health interventions also, regardless of clinical diagnoses.

Providing intervention to support infant mental health before mental health difficulties are evident will ensure a future of thriving and strong infants, children and adolescents who will mature into healthy adults (Weitzman et al., 2015). Whilst this model shows promise, further updating to improve its PPV, external validation, as well as local validation and testing the feasibility, acceptability and usability of it, are next steps before implementation and to deem it clinically useful.

### Strengths and limitations

The use of a population‐based sample for model development is a key strength which enhances generalisability. As is applying PROBAST, calculating sample size a priori and using predictors identified in a different cohort for child mental health. Correcting for optimism and providing (in)stability metrics provides reassurance that the model is reliable.

Gestational age‐range of the included sample was from 30‐weeks+. As a result, this model cannot be applied to children born <30‐weeks’ gestation.

Restricting to complete‐case analysis rather than carrying out multiple imputation is a potential limitation. However, using incorrectly specified models for multiple imputation would arguably increase bias.

ALSPAC is predominantly a white cohort and thus generalisability of findings to other races is extremely limited and this work would need to be conducted in a more diverse cohort. The known selection bias in longitudinal studies favouring people with less adverse circumstances continuing with on‐going follow‐up is also a limitation. As most predictors were measured based on self‐report, there is a possibility of measurement bias. Whilst evaluating one's own prediction model is a useful first step, an independent evaluation conducted by authors not involved in the development is required.

This data is from a middle‐high resource setting and so the model would need to be replicated using data from low‐resource setting. Finally this cohort was born in the 1990s and generalisability to more contemporary groups could be problematic however we showed very similar findings in a French cohort at 5‐years born in 2011 which is reassuring.

### Conclusion

Our findings show that it is possible to predict poor mental health at 7‐years using information known in the perinatal period. 85.6% of the participants mental health at 7‐years was accurately predicted in the perinatal period before mental health symptoms would be evident. As is known where the outcome prevalence is low, it performed better for predicting those who do not later exhibit poor mental health. Our model showed good classification performance, calibration and fairness across our groups of interest (sex, sociodemographic risk and time spent in SCBU). This prediction model was generated using only non‐invasive, low‐cost information making it easier to implement in clinical practice as it incorporates information that can be self‐reported and aligns with routine developmental check appointments in the early years.

## AUTHOR CONTRIBUTIONS

**Emma Butler**: Conceptualization; formal analysis; methodology; software; visualization; writing original and writing review. **Michelle Spirtos**: Conceptualization; methodology; supervision; writing review. **Linda M. O'Keeffe**: Conceptualization; methodology; supervision; writing review. **Mary Clarke**: Conceptualization, methodology, supervision, writing review.

## CONFLICT OF INTEREST STATEMENT

Dr. L.M.O. is funded by a Health Research Board of Ireland Emerging Investigator Award (grant ref: EIA‐FA‐2019‐007 SCaRLeT). The remaining authors have declared that they have no competing or potential conflicts of interest.

## ETHICAL CONSIDERATIONS

Ethical approval for the study was obtained from the ALSPAC Ethics and Law Committee and the Local Research Ethics Committees. Informed consent was appropriately obtained for the use of data collected via questionnaires and clinics was obtained from participants following the recommendations of the ALSPAC Ethics and Law Committee at the time. This secondary data analysis was approved on 12/01/2023 by the Research Ethics committee for the Royal College of Surgeons Ireland (RIMS number 212610659).

## Supporting information

Supporting Information S1

## Data Availability

Data available: https://www.bristol.ac.uk/alspac/researchers/our‐data/.
