# Supplementary material for: Predicting 7‐year‐olds mental health in the perinatal period: Development and internal validation of a multivariable model using the prospective ALSPAC cohort
Source: JCPP Adv. 2026 Jan 3:e70091. Online ahead of print. doi: 10.1002/jcv2.70091 (PMC13339273; doi:10.1002/jcv2.70091)
Supplement: Supplementary file 1 — Supporting Information S1 [file JCV2-9999-e70091-s001.docx]

**Predicting 7-year-olds mental health in the perinatal period: development and internal validation of a multivariable model**

**using the prospective ALSPAC cohort**

**Supporting Information**

**Table S1: sample size calculation**

For our binary outcome at 7-years, we have 468/8351 (304/6021) with the event. Therefore, the following sample size calculations apply:

| Assumed C-statistic | Inputs | Events Per predictor parameter (EPP) |
| --- | --- | --- |
| 0.84 | 67 (60) | 6.98 (5.12) |
| 0.74 | 40 (26) | 11.69 (11.82) |
| 0.70 | 27 (18) | 17.32 (17.06) |
| 0.64 | 12 (8) | 38.97 (38.38) |

Aiming for a modest C-statistic, 0.7 we will input a maximum of (18) ***candidate*** predictors. It is important to note that each *level* of a factor variable is considered one input.

Note: no bracket=calculation based on ‘n’ that participated at follow-up prior to cleaning data. Bracketed=calculation conducted after determining sample size of complete cases.

**Table S2: candidate predictors and outcome in ALSPAC**

| Variable | ALSPAC File location/time measured | Question/Measure | Response |  |
| --- | --- | --- | --- | --- |
| Predictors: | | | |  |
| *Pre-pregnancy health* | | | |  |
| Gravidity | B002 ~18weeks gestation | Were you previously pregnant? | Binary N/Y (65%yes) |  |
| Maternal mental health | D167a/168a/  169a/170a/  171a/172a ~ 12weeks gestation | If endorsed any of EVER:  drug addiction, alcoholism, schizophrenia, anorexia, severe depression, ‘other’ psychiatric problem | Binary N/Y (10% yes) |  |
| History of blood pressure problems before current pregnancy? | D046/047 ~ 12weeks gestation | 1815 reported a history of hypertension (1329 of whom was in a prior pregnancy) – both responses coded as yes history of blood pressure problems | Binary N/Y (14% yes) |  |
| *During pregnancy:* | | | |  |
| Pregnancy complications (**biological** pregnancy specific experiences) | B044/C052/E100  B046/C053/E101  B048/C054/E102  B050/C055/E103  B054/C057/E105  B056/C059/E106  B058/C060/E107  B060/C061/E108  B062/C062/E109  B063/C063/E110  B068/C066/E112  pregnancy_diabetes  prev_hyp/HDP  E116 ~18 weeks gestation, ~32 weeks gestation, ~8 weeks postnatal | 1 point was given for each of the below if endorsed at ANY time during the pregnancy (ie ~18 weeks asked in trimester 1, ~32 weeks asked in trimester 2, ~8 weeks post-natal asked in trimester 3):  Nausea, vomiting, diarrhea, bleeding, UTI, Flu, Rubella, thrush, herpes, ‘other’ infection, injury/shock, diabetes (existing, gestational or glycosuria), HBP (existing or HBP after 20 wks gestation), ‘other’ complication | Max total was 14 but range in sample was from 0-10.  0 – 4%  1 – 11%  2 – 21%  3 – 25%  4 – 20%  5 – 12%  6 – 5%  7 – 2%  8 - .5%  9 - .08%  10 - .03%  This was then amalgamated into a four level variable ranging from 0-3+  0- 16% (0&1)  1- 65% (2,3 &4)  2- 17% (5 & 6)  3 - 2% (7-10) |  |
| Psychosocial pregnancy specific experiences | mlon011- Family adversity index in pregnancy ~8, 12, 18 & 32 weeks gestation  (Winsper et al., 2012)  10.1017/  S0033291712000542 | See Winsper et al 2012 for how this variable was constructed (range 0-11), it was then categorised by the original authors:0=no adversity, score of 1+2=mild adversity, score of 3+=severe adversity | None-47%  Mild -40%  Severe-13% |  |
| Smoking in pregnancy | A200  (A file asked during pregnancy ranging from 8-42 weeks gestation) | Number of cigarettes smoked presently (dichotomised as none v any) | Binary N/Y (15%yes) |  |
| Alcohol in 1^st^ trimester of pregnancy | B721 ~18 weeks gestation | How much alcohol did you drink in first 3months of pregnancy (dichotomised as none v any) | Binary N/Y (56%yes) |  |
| *Birth/postnatal:* | | | |  |
| Special Care Baby Unit | ka014 ~ 4 weeks postnatal | Baby was admitted to special care baby unit? | Binary N/Y (5% yes) |  |
| Sex of infant |  | Participant assigned sex at birth | 0-male, 1-female  51% male |  |
| Post-natal sociodemographic risk | kaTownsendq5 ~ 4 weeks postnatal  c800 ~ 32 weeks gestation  c645a ~ 32 weeks gestation  A form a520 – between 8 and 42 weeks gestation  A form a901 – between 8 and 42 weeks gestation | Income quintile when child is 1month old  Ethnicity/race  Highest level maternal education (last trimester/early postnatal)  Do you have a partner?  Maternal age on completion of questionnaire | Top 2 quintiles coded as 0: 46%, middle as 1: 21%, bottom 2 quintiles as 2: 34%  99% coded as 0 (nonhispanic white), 1% as 1 (other)  Degree coded as 0: 16% A-level coded as 1: 26%, O level & vocational coded as 2: 45% and none/CSE coded as 3: 13%  Binary N/Y (1.3% said no – coded as 1)  <26 years & >35 years coded as 1: 28%, rest coded as 0: 72%  These 5 variables were then summed and total social risk score categorised as:  None – 20%  Low – 49%  Mod -28%  High – 3% |  |
| Outcome: | | | | |
| strengths and difficulties questionnaire (SDQ) total score @ 7-years | | kq348f ~6y9m (81 months) | Total continuous SDQ score/40 but dichotomised as clinical or not based on cut-offs >16 | Binary not clinical/clinical level (5.1% clinical) |

**Table S3: univariate associations between each candidate predictor and outcome n=6021**

| Predictor | Odds Ratio (95% Confidence Interval) | P-value | Coefficient (95% Confidence Interval) |
| --- | --- | --- | --- |
| Gravidity (Ref Not pregnant before) |  |  |  |
| Yes pregnant before | .9 (.7-1.2) | .55 | -.07 (-.31-.17) |
|  |  |  |  |
| Maternal mental health problems (Ref no) |  |  |  |
| yes | 2.5 (1.9-3.3) | .000 | .91 (.63-1.2) |
|  |  |  |  |
| History of high blood pressure (Ref no) |  |  |  |
| Yes outside a pregnancy &/or during a previous pregnancy | 1.1 (.8-1.5) | .72 | .06 (-.27-.39) |
|  |  |  |  |
| Number biological ^a^PSEs (Ref none) |  |  |  |
| 1 | 1.2 (0.9-1.8) | .25 | .21 (-.15-.57) |
| 2 | 1.8 (1.2 – 2.7) | .00 | .57 (.15-.98) |
| 3+ | 2.0 (.9-4.3) | .07 | .69 (-.06-1.45) |
|  |  |  |  |
| Psychosocial PSEs (Ref low): | | | |
| Mild | 1.6 (1.2-2.1) | .001 | .45 (.17-.73) |
| Severe | 3.9 (2.9-5.3) | .00 | 1.37 (1.1-1.7) |
|  |  |  |  |
| Smoked in this pregnancy | 1.9 (1.5-2.5) | .000 | .66 (.39-.93) |
|  |  |  |  |
| Drank alcohol in 1^st^ trimester in this pregnancy | 1.1 (.9-1.4) | .32 | .12 (-.12- .35) |
|  |  |  |  |
| Special Care Baby Unit (SCBU) | 1.3 (.8-2.1) | .27 | .26 (-.21-.73) |
|  |  |  |  |
| Sex (Ref male) |  |  |  |
| Female | .7 (.5-.9) | .001 | -.38 (-.62- -.15) |
|  |  |  |  |
| No sociodemographic risk (Ref) |  |  |  |
| Low | 1.8 (1.2-2.6) | .002 | .59 (.22-.96) |
| Moderate | 2.3 (1.5-3.4) | .000 | .82 (.43-1.21) |
| High | 3.6 (1.9-6.7) | .000 | 1.27 (.64-1.90) |

^a^Pregnancy-specific-experiences

**Fig S1: participant flow-chart**

14633 fetuses recruited in original core sample Phase 1

(13988 of who were alive at 1-year)

8351 took part in the follow-up wave at 7-years

(ie had the SDQ outcome)

6021 had full data available (ie 72.1% of those who had the outcome were included for complete case analysis)

**Table S4: PROBAST tool for risk of bias and applicability of the ALSPAC dataset.**

| Participants: | |
| --- | --- |
| Were appropriate data sources used? | Yes population level pregnancy cohort so matches intended population and time-point of use |
| Were all inclusions & exclusions of participants appropriate? | 14633 fetuses (13988 alive at 1-year) – 8351 = 6282 excl as didn’t participate in wave with outcome. (yes)  8351-6021=2330 with some missing (No could have performed multiple imputation). |
| Predictors: | |
| Were predictors defined & assessed in similar way for participants in all clusters? | yes |
| Were predictor assessments made without knowledge of outcome data? | Yes prospectively at all maternity sites |
| Are all predictors available at the time the model is intended to be used? | Yes all birth model known by 1-months, all infancy model (to see if motor prognostic) known by 18-months. |
| Outcome: | |
| Was the outcome determined appropriately? | Yes as per recommended scoring |
| Was a pre-specified or standard outcome definition used? | Yes at clinical cut offs |
| Were predictors excluded from the outcome definition? | Yes |
| Was the outcome defined and determined in a similar way for all participants? | Yes, the total score was calculated the same way for all participants. |
| Was the outcome determined without knowledge of predictor information? | Yes |
| Was the time interval between predictor assessment & outcome determination appropriate? | Yes 7 years for birth model. |
| Analysis: | |
| Were there a reasonable number of participants with the outcome? | Yes (n=304, 5.1%) and sample size calculated a priori and again once data analysis and final sample size was known. |
| Were continuous and categorical predictors handled appropriately? | Yes. All predictors were categorical . |
| Were all enrolled participants included in analysis? | No |
| Were participants with missing data handled appropriately? | Possibly no, could have performed Multiple Imputation for the 28% excluded. |
| Was selection of predictors based on univariable analysis avoided? | Yes predictors based on predictors in model in different dataset and used LASSO for predictor selection within that. |
| Were complexities in the data (e.g. competing risks) accounted for appropriately? | Probably no as we had no way of knowing which participants availed of intervention between aged birth and 7y. |
| Were relevant model performance measures evaluated appropriately? | Yes |
| Were model over-fitting, under-fitting and optimism in model performance accounted for? | Yes |
| Do predictors and their assigned weights in the final model correspond to the results from the reported multivariable analysis? | Yes? |
| Overall applicability | No concerns |
| Overall risk of bias | High (ie low for predictors and outcome, but high for participants and analysis due to not imputing for missing) |

**Table S5: case-mix distribution between original developed model in ELFE and new developed model in ALSPAC**

| Variable | ELFE @ 5-years  In 2011 | ALSPAC@ 7-years  In 1990 |
| --- | --- | --- |
| Clinical SDQ^a^ (%) | 6.1 | 5.1 |
| SDQ-total (mean(SD)) | 8.4 (4.7) | 7.4 (4.7) |
| SDQ-total (median(IQR)) | 8 (5-11) | 7 (4-10) |
| SDQ range | 0-33 | 0-31 |
| Cumulative sociodemographic risk: |  |  |
| None | 31.5 | 20.4 |
| Low | 29.5 | 49.2 |
| Moderate | 32.2 | 27.8 |
| High | 6.8 | 2.6 |
| Maternal mental health problems prior to pregnancy (%yes) | 23.1 | 10.4 |
| Maternal blood pressure problems outside of pregnancy (%yes) | 2.7 | 13.8 |
| Gravidity ie previously pregnant (%yes) | 70.8 | 65.4 |
| Cumulative biopsychosocial pregnancy experiences (%) |  |  |
| 0 | 34.4 | 8.5 |
| 1 | 33.5 | 36.6 |
| 2 | 18.4 | 34.7 |
| 3 | 8.7 | 15.9 |
| 4+ | 5.0 | 4.3 |
| Male (%) | 50.1 | 51.2 |
| Smoked in pregnancy (% yes) | 19.5 | 14.8 |
| Alcohol in pregnancy (% yes) | 24.0 | 56.1 |
| NICU^b^/SCBU^c^ (%) | 5.2 | 5.3 |
| Gestational age – weeks (mean(SD)) | 39.3 (1.4) | 39.6 (1.6) |
| Gestational age - weeks (range) | 32-42 | 30-42 |

^a^Strengths and Difficulties Questionnaire total score. ^b^Neonatal Intensive Care Unit. ^c^Special care baby unit.

**Table S6a: comparing those included in the analysis (n=6021) with those excluded due to missing outcome or predictor (n=8612)**

| Variable | Whole sample  (n=14633) | Excluded  (n=8612 (59%)) | Included  (n=6021(41.2%)) |
| --- | --- | --- | --- |
| Gravidity (yes) | 8906 (67%)  (n=13284) | 4967 (68.4%)  (n=7263) | 3939 (65.4%) |
| Maternal mental health problems | 1511(12%)  (n=12548) | 883 (13.5%)  (n=6527) | 628 (10.4%) |
| History of high blood pressure | 1815 (14.8%)  (n=12295) | 985 (15.7%)  (n=6274) | 830 (13.8%) |
| Biological ^a^PSEs: | (n=9907) | (n=3886) |  |
| 0 | 1495 (15.1%) | 554 (14.3%) | 941 (15.6%) |
| 1 | 6480 (65.4%) | 2516 (64.8%) | 3964 (65.8%) |
| 2 | 1702 (17.2%) | 705 (18.1%) | 997 (16.6%) |
| 3+ | 230 (2.3%) | 111 (2.9%) | 119 (2.0%) |
| Psychosocial PSEs: | (n=13290) | (n=7269) |  |
| None | 5316 (40%) | 2503 (34.4%) | 2813 (46.7%) |
| Mild | 5540 (41.7%) | 3135 (43.1%) | 2405 (39.9%) |
| Severe | 2434 (18.3%) | 1631 (22.4%) | 803 (13.3%) |
|  |  |  |  |
| Smoked in pregnancy (yes) | 3626 (26.6%)  (n=13632) | 2738 (36%)  (n=7611) | 888 (14.8%) |
| Alcohol in 1^st^ trimester in pregnancy (yes) | 7155 (54.5%)  (n=13121) | 3775 (53.2%)  (n=7100) | 3380 (56.1%) |
| Special Care Baby Unit (yes) | 865 (7.1%)  (n=12201) | 550 (8.8%)  (n=6223) | 315 (5.3%)  (n=5978) |
| Infant sex (female) | 6820 (48.3%)  N=14118 | 3883 (48%) | 2937 (48.8%) |
| Post-natal sociodemographic risk: | (n=10378) | (n=4357) |  |
| None | 1741 (16.8%) | 514 (11.8%) | 1227 (20.4%) |
| Low | 4751 (45.8%) | 1789 (41.1%) | 2962 (49.2%) |
| Moderate | 3321 (32%) | 1647 (37.8%) | 1674 (27.8%) |
| High | 565 (5.4%) | 407 (9.3%) | 158 (2.6%) |
| SDQ* (clinical) | 468 (5.6%)  N=8351 | 164 (7.0%)  N=2330 | 304 (5.1%) |
| SDQ* – mean (SD) range | 7.6(4.9)  0-33  N=8351 | 8.0(5.1)  0-33  N=2330 | 7.4(4.7)  0-31 |

^a^Pregnancy-specific-experiences. *whole sample is the 8351 who took part at 7-years and thus have Strengths and Difficulties (SDQ) outcome data but n=2330 were excluded from analysis as they did not have complete predictor data.

**Table S6b: distribution of missing predictors within the 8351 who had the outcome as they took part in the 7-year follow-up wave.**

| Number of variables missing | % of cases (n) |
| --- | --- |
| None | 72.1 (6021) |
| One | 10.8 (902) |
| Two | 10.7 (890) |
| Three | 3.0 (252) |
| Four | 1.5 (126) |
| Five | 0.7 (62) |
| Six | 0.3 (25) |
| Seven | 0.6 (50) |
| Eight | .01 (1) |
| Nine | 0.3 (22) |

**Table S6c: patterns of missing data for the 8351 who took part at 7-year but excluding scbu as that wasn’t needed for model development:**

| N= | Variables missing |
| --- | --- |
| 6021 | Miss nothing |
| 629 | Only missing cumulative sociodemographic risk |
| 338 | Only missing smoking and biological Pregnancy Specific Experiences (PSEs) |
| 311 | Only missing motor information ie not partake at 18m |
| 180 | Only missing biological PSEs |
| 139 | Only missing sociodemographic risk and biological PSEs |
| 87 | Only missing previous blood pressure and biological PSEs |
| 86 | Missing smoke, sociodemographic risk and biological PSEs |

Note: The rest of the missing patterns are less than n=50.

**Table S7: model performance statistics for the model after internal validation using bootstrapping.**

| Model: | Performance | | |
| --- | --- | --- | --- |
| Statistic | Original apparent | Optimism | Optimism adjusted |
| C-statistic | 0.67 (0.64-0.70) | -.018 | 0.66 (0.64-0.68) |
| Calibration-in-the-large (CITL) | 0 (-0.00) | .001 | .001 (-.17-.24) |
| C-slope | 1 (1.02) | -.095 | 0.93 (.71-1.2) |

Note: There was minimal optimism adjustment to the C-statistic indicating good internal performance in terms of discrimination. The mis-calibration in CITL is very small at <1% basically zero miscalibration in overall risks. The calibration slope suggests a moderate amount of shrinkage is required to adjust the predictor effects in the model ie over fitted (predictions are too extreme), driven by under-prediction in those with estimated event probabilities above 0.2 (see calibration plot).

**Table S8: distribution of the linear predictor (histograms) from the model for those with and without poor mental health.**

|  | Adequate mental health (n=5717) | Poor mental health  (n=304) |
| --- | --- | --- |
| Mean linear predictor (LP) | -3.11 | -2.73 |
| Median LP | -3.16 | -2.88 |
| SD LP | 0.55 | 0.65 |
| IQR LP | -3.46- -2.82 | -3.20- -2.26 |

**Fig S2: distribution of the linear predictor for those with (blue) and without (red) adequate mental health**


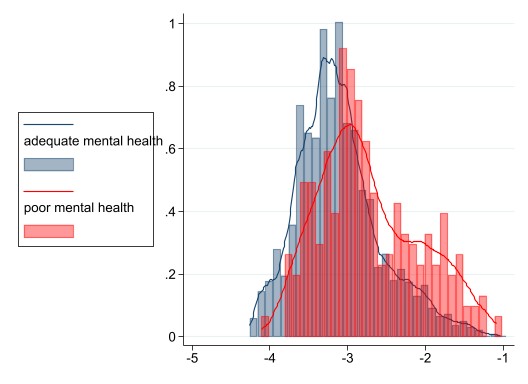


**Fig S3: histogram of the predicted probability values for the sample from the prediction model.**


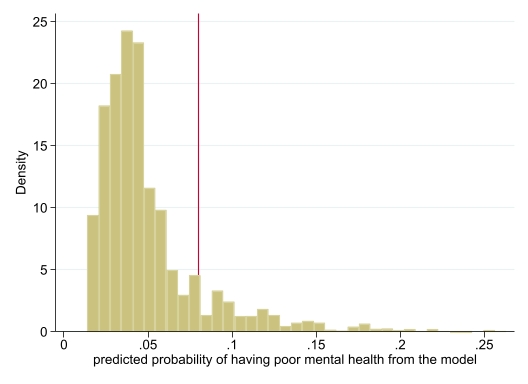


Note: No child has predicted risk higher than .26, min is 0.02, median 0.04 and red line indicates our threshold at 0.08.

**Fig S4a: calibration instability plot**


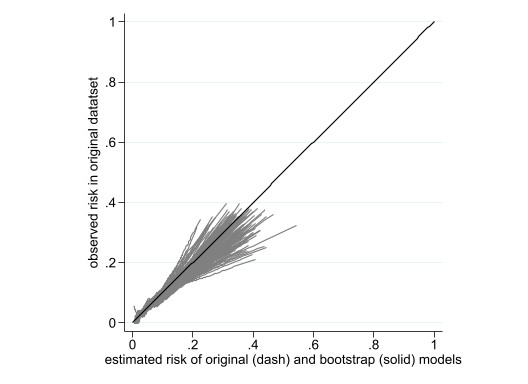


**Fig S4b: classification plot**


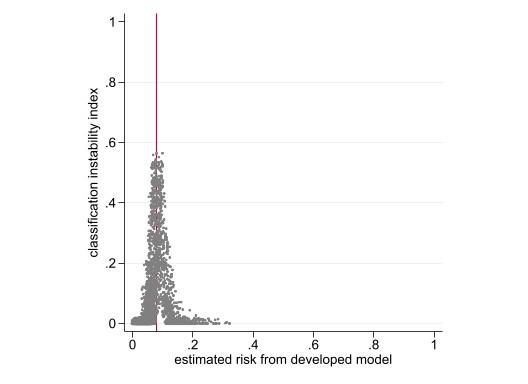


**Fig S4c: c-statistic instability histogram**


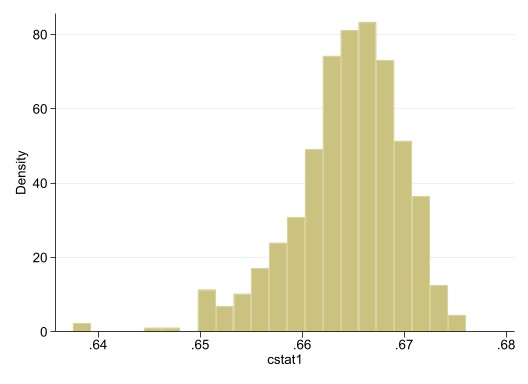


**Fig S4d: summary statistics for the mean absolute prediction error (MAPE)**

**Fig S5: classification based on 8% risk threshold of predicted with observed**

**
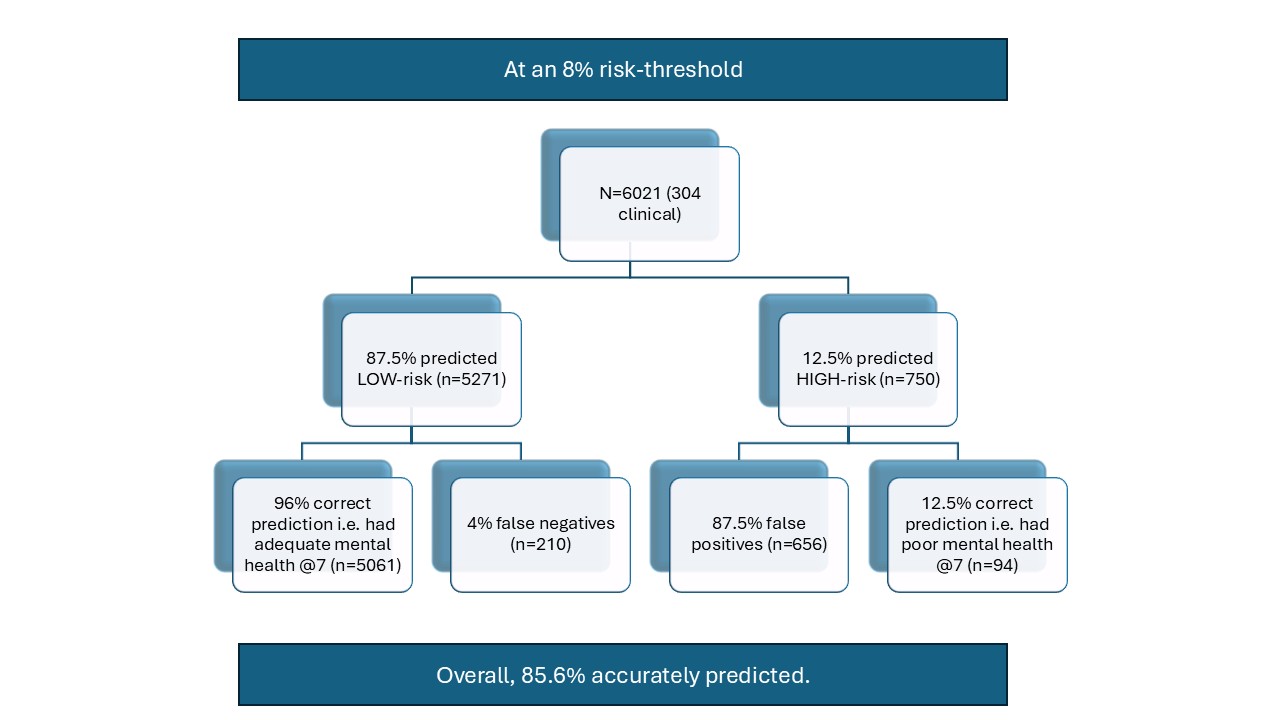
**

**Fig S6: decision curve analysis**


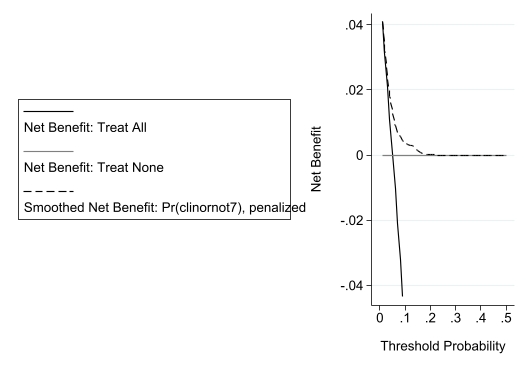


Note: Decision curves showing net benefit for binary prediction model of poor mental health across a range of threshold probabilities. threshold probability = risk needed to initiate a clinical action. Positive values of net benefit indicate clinical utility. Treat all = strategy of initiating a clinical action for all patients regardless of their estimated risk. Treat none = strategy of not initiating a clinical action for any patient. Dashed line = strategy of initiating a clinical action for those patients whose estimated risk is at or above the threshold probability.

**Table S9: The proportion of children with poor mental health at 7-years examined across sex, social risk and Special Care Baby Unit (SCBU)**

| Sex: | % poor mental health (95%CI) |
| --- | --- |
| Male (n=3084) | .06 (.05-.07) |
| Female (n=2937) | .04 (.03-.05) |
| Cumulative sociodemographic risk: |  |
| None (n=1227) | .03 (.02-.04) |
| Low (n=2962) | .05 (.04-.06) |
| Moderate (n=1674) | .06 (.05-.08) |
| High (n=158) | .10 (.05-.15) |
| SCBU: |  |
| No SCBU (n=5663) | .05 (.04-.06) |
| Yes SCBU (n=315) NB only ≥30weeks gestation | .06 (.04-.10) |
| Missing SCBU (n=43) | .07 (.01-.19) |

**Table S10: comparing the model performance in our different subgroups of interest (sex, sociodemographic risk and Special Care Baby Unit (SCBU)).**

| Group | N= | ROC area | Standard Error | 95% CI |
| --- | --- | --- | --- | --- |
| All | 6021 | 0.67 | 0.016 | 0.64-0.70 |
| Sex |  |  |  |  |
| Male | 3084 | 0.65 | 0.02 | 0.61-0.69 |
| Female | 2937 | 0.68 | 0.03 | 0.63-0.73 |
| Sociodemographic Risk |  |  |  |  |
| None | 1227 | 0.68 | 0.04 | 0.60-0.76 |
| Low | 2962 | 0.64 | 0.02 | 0.59-0.68 |
| Moderate | 1674 | 0.67 | 0.03 | 0.61-0.72 |
| High | 158 | 0.77 | 0.06 | 0.65-0.89 |
| SCBU |  |  |  |  |
| No | 5663 | 0.68 | 0.02 | 0.64-0.71 |
| Yes | 315 | 0.57 | 0.07 | 0.44-0.70 |

**Table S11: co-efficient values for predictor variables when the fine-motor and gross-motor Z-score variables were included as possible candidate predictors**

| Predictors | ^a^Model Coefficient |
| --- | --- |
| Intercept | -3.80 |
| ^b^Level of biological pregnancy-specific: |  |
| 0 | Ref |
| 1 | .14 |
| 2 | .41 |
| 3 | .28 |
| ^c^Level of psychosocial pregnancy-specific: | |
| None | Ref |
| Mild | .31 |
| Severe | 1.10 |
| ^d^Cumulative sociodemographic risk: |  |
| None | Ref |
| Low | .47 |
| Moderate | .52 |
| High | .63 |
| History of psychological difficulties before being pregnant: |  |
| No | Ref |
| Yes | 0.49 |
| Pregnant before | -0.18 |
| Smoked during the pregnancy: |  |
| No | Ref |
| Yes | 0.19 |
| Consumed alcohol during the pregnancy: |  |
| No | Ref |
| Yes | 0.05 |
| Infant sex: |  |
| Male | Ref |
| Female | -0.25 |
| Fine-motor | -0.36 |
| Performance and discrimination metrics: | |
| Discrimination |  |
| AUC | 0.70 (0.67-0.73) |

**Table S12: logistic regression of predicting (a) poor mental health at nine-years and (b) poor mental health at 11-years-old using the babies predicted risk group (i.e. predicted to be low- or high-risk of later poor mental health) applying an 8% risk threshold cut-off.**

|  | **Odds Ratio** | **Standard Error** | **z** | **95% confidence interval** |
| --- | --- | --- | --- | --- |
| 1. @9-years-old (N=5098) | | | | |
| High-risk  (ref low-risk) | 3.57 | 0.57 | 7.94 | 2.61-4.88 |
| Constant | .03 | .00 | -40.56 | .03-.04 |
| 1. @11-years-old (N=4710) | | | | |
| High-risk  (ref low-risk) | 2.98 | .50 | 6.46 | 2.14-4.15 |
| Constant | .04 | .00 | -39.73 | .03-.04 |

**Table S13: the accuracy of categorisation of children as predicted ‘high’ or ‘low-risk’ of poor mental health (SDQ-total >16) at 7-years compared to their observed outcome based on (1) predicted values based on a model using cumulative sociodemographic risk as the only predictor (2) predicted values based on a model using history of maternal mental health problems as the only predictor (3) predicted values based on the risk prediction model discussed in this paper. Please note all groups used a risk threshold based on ≥8% risk-threshold as the cut-point to define ‘low’ and ‘high’ risk.**

| Proportion identified at high-risk  % (n) | Predicted ‘low-risk’ group  % (n) | | Predicted ‘high-risk’ group  % (n) | |
| --- | --- | --- | --- | --- |
|  | Observed Adequate mental health | Observed Poor mental health | Observed Adequate mental health | Observed Poor mental health |
| **@8% risk-threshold (1) cumulative sociodemographic risk**  (Accurately identified **4.9%** of those who experienced poor mental health at birth) | | | | |
|  | N=5863 | | N=158 | |
| 2.6 (158) | 95.1 (5574) | 4.9 (289) | 90.5 (143) | 9.5 (15) |
| **@8% risk-threshold (2) history of maternal mental health difficulties**  (Accurately identified **21.4%** of those who experienced poor mental health at birth) | | | | |
|  | N=5393 | | N=628 | |
| 10.4 (628) | 95.6 (5154) | 4.4 (239) | 89.7 (563) | 10.3 (65) |
| **@8% risk-threshold (3) risk prediction model discussed in the paper**  (Accurately identified **30.9%** of those who experienced poor mental health at birth) | | | | |
|  | N=5271 | | N=750 | |
| 12.5 (750) | 96.0 (5061) | 4.0 (210) | 87.5 (656) | 12.5 (94) |

**Fig S7: ELFE prediction model predictors and coefficients (purple) and ALSPAC prediction model predictors and coefficients (black)**


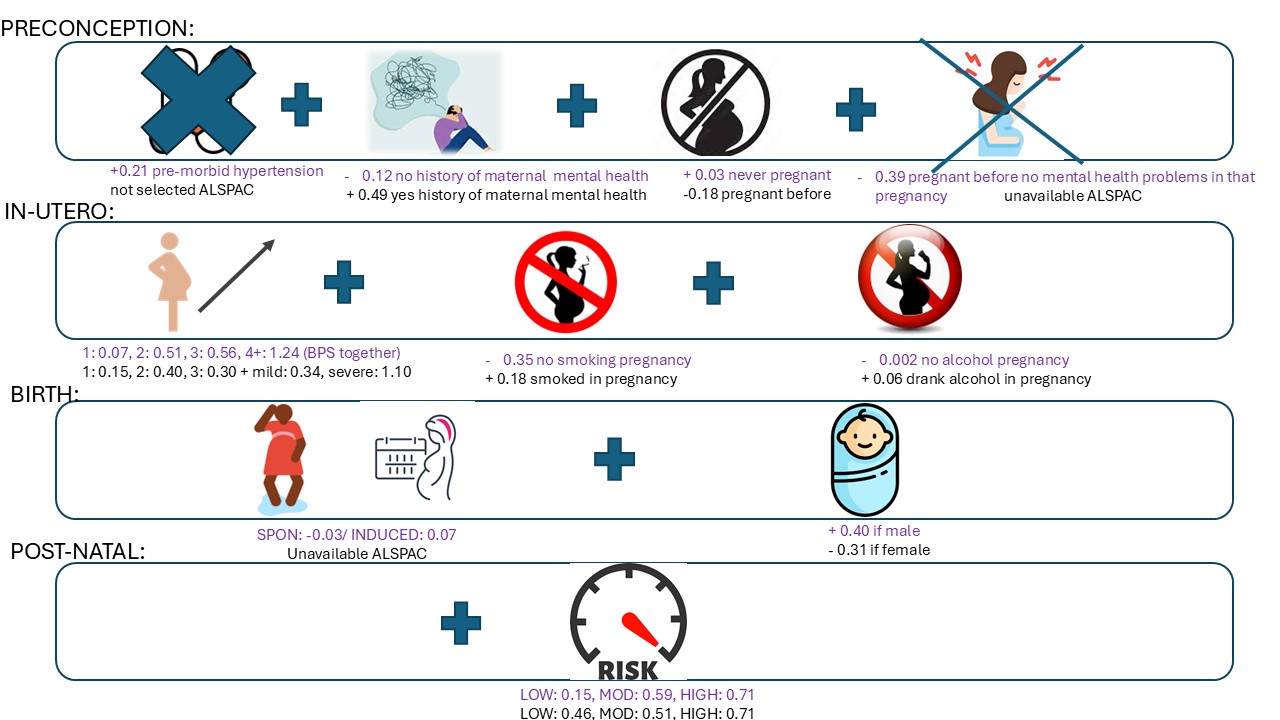


**Table S14: illustrating the impact of differing risk-thresholds of 25%, 15% and 8% cut-offs on over- and under-predicting risk in subgroups of interest. Poor mental health defined by total-SDQ >16.**

|  |  | **OBSERVED** | **@ 25% cut** | **@ 15% cut** | **@ 8% cut** |
| --- | --- | --- | --- | --- | --- |
| Special Care Baby Unit | No | 95% adequate mental health  5% poor mental health(n=281) | 99.9% not predicted to be HR*.  0.1% (n=8) predicted to be HR. | 97.8% not predicted to be HR.  2.2% predicted to be HR (n=126) | 87.8% not predicted to be HR.  12.2% predicted to be HR. (n=692) |
|  | |  |  |  |  |
|  | Yes | 93.7% adequate mental health  6.3% poor mental health (n=20) | 100% not predicted to be HR.  N=315. | 95.6% not predicted to be HR.  4.4% predicted to be HR. (n=14) | 83.8% not predicted to be HR.  16.2% predicted to be HR (n=51) |
|  | |  |  |  |  |
| Predictions:  Over/under, too low/high | | | Under, too low | Under, too low | Over, too high. |
| Sex | Male | 94.1% adequate mental health  5.9% poor mental health (n=183) | 99.7% not predicted to be HR.  0.3% (n=8) predicted to be HR. | 96.3% not predicted to be HR.  3.7% predicted to be HR (n=113) | 84.4% not predicted to be HR.  15.6% predicted to be HR. (n=480) |
|  | |  |  |  |  |
|  | Female | 95.9% adequate mental health  4.1% poor mental health (n=121) | 100% not predicted to be HR. | 99% not predicted to be HR.  1% predicted to be HR. n=29 | 90.8% not predicted to be HR.  9.2% predicted to be HR. (n=270) |
|  | |  |  |  |  |
| Predictions:  Over/under, too low/high | | | Under, too low | Under, too low | Over, too high. |
|  |  | **OBSERVED** | **@ 25% cut** | **@ 15% cut** | **@ 8% cut** |
| History of maternal mental health difficulties | No | 95.6% adequate mental health  4.4% poor mental health (n=239) | 100% not predicted to be HR. | 99.6% not predicted to be HR.  0.4% predicted to be HR (n=21) | 91.8% not predicted to be HR.  8.2% predicted to be HR. (n=444) |
|  | |  |  |  |  |
|  | Yes | 89.7% adequate mental health  10.3% poor mental health (n=65) | 98.7% not predicted to be HR.  1.3% predicted to be HR.  N=8. | 80.7% not predicted to be HR.  19.3% predicted to be HR. n=121 | 51.3% not predicted to be HR.  48.7% predicted to be HR (n=306) |
|  | |  |  |  |  |
| Predictions:  Over/under, too low/high | | | Under, too low | Under, too low if no maternal history and over, too high in yes maternal history | Over, too high. |
| Cumulative  Sociodemographic risk | None | 97.2% adequate mental health  2.8% poor mental health (n=35) | 100% not predicted to be HR. | 100% not predicted to be HR. | 98.8% not predicted to be HR.  1.2% predicted to be HR. (n=15) |
|  | |  |  |  |  |
|  | Low | 95% adequate mental health  5% poor mental health (n=149) | 99.9% not predicted to be HR.  0.03% predicted to be HR  N=1. | 98.5% not predicted to be HR.  1.5% predicted to be HR. n=46 | 89.7% not predicted to be HR. |
|  | |  |  |  | 10.3% predicted to be HR (n=304) |
|  | Mod | 93.7% adequate mental health  6.3% poor mental health (n=105) | 99.7% not predicted to be HR.  0.3% predicted to be HR. n=5 | 95.6% not predicted to be HR.  4.4% predicted to be HR. n=73 | 78.8% not predicted to be HR.  21.2% predicted to be HR. n=355 |
|  | High | 90.5% adequate mental health  9.5% poor mental health (n=15) | 98.7% not predicted to be HR.  1.3% predicted to be HR.  N=2 | 85.4% not predicted to be HR.  14.6% predicted to be HR. n=23 | 51.9% not predicted to be HR.  48.1% predicted to be HR. n=76 |
| Predictions:  Over/under, too low/high | | | Under, too low | Under, too low in all groups except for high sociodemographic risk where over, too high. | Under, too low in no sociodemographic risk group while Over, too high in all other groups |

*HR=high-risk
